# A Fulminant Case of Demyelinating Encephalitis With Extensive Cortical Involvement Associated With Anti-MOG Antibodies

**DOI:** 10.3389/fneur.2020.00031

**Published:** 2020-02-14

**Authors:** Sonja Hochmeister, Thomas Gattringer, Martin Asslaber, Verena Stangl, Michaela Tanja Haindl, Christian Enzinger, Romana Höftberger

**Affiliations:** ^1^Department of Neurology, Medical University Graz, Graz, Austria; ^2^Department of Pathology, Medical University Graz, Graz, Austria; ^3^Division of Neuroradiology, Vascular and Interventional Radiology, Department of Radiology, Medical University Graz, Graz, Austria; ^4^Division of Neuropathology and Neurochemistry, Department of Neurology, Medical University of Vienna, Vienna, Austria

**Keywords:** MOG, autoimmunity, antibodies, encephalitis, demyelination

## Abstract

Anti-myelin oligodendrocyte glycoprotein (MOG) antibodies (MOG-Abs) are commonly associated with clinical presentations as acute disseminated encephalomyelitis (ADEM) in both adults and children and anti-aquaporin 4 antibody-seronegative neuromyelitis optica spectrum disorder (NMOSD) and related syndromes such as optic neuritis, myelitis, and brainstem encephalitis. Most often, the presence of MOG-Abs is associated with a more benign clinical course and a good response to steroids. Here, we present a case report of a previously healthy 52-year-old female patient with fulminant demyelinating encephalitis, leading to death within a week after the first presenting symptoms from a massive brain edema irresponsive to high-dose intravenous steroids as well as osmotic therapy. The final diagnosis was only made postmortem after serum anti-MOG-Abs results were available. Histopathological analysis of the brain revealed extensive, predominantly cortical demyelinating lesions in the frontal, temporal, and parietal lobes with intracortical, leukocortical, and subpial plaques, associated with pronounced perivenous deposition of activated complement complex as well as features of acute MS characterized by destructive lesions.

## Case Study

### Clinical Course

A 52-year-old woman, a cashier at a supermarket, presented in our outpatient department with difficulties in speaking. Furthermore, she complained of headache in the last 2 weeks. At neurological examination, she showed features of an encephalitic syndrome (disorientation, agitation, poor memory functioning) and continuously perseverated the same sentences. Gaze was preferable to the right, but there were no motor signs on admittance. Meningeal signs were positive. Past medical history was remarkable for hypothyreodism, a previous gastric-banding surgery in 2011, and depression. She was on a permanent antidepressive medication (Sertralin/Trazodon) as well as thyroid hormone replacement. Allergies were documented against Diclofenac and Penicillin.

**Figure 1:**
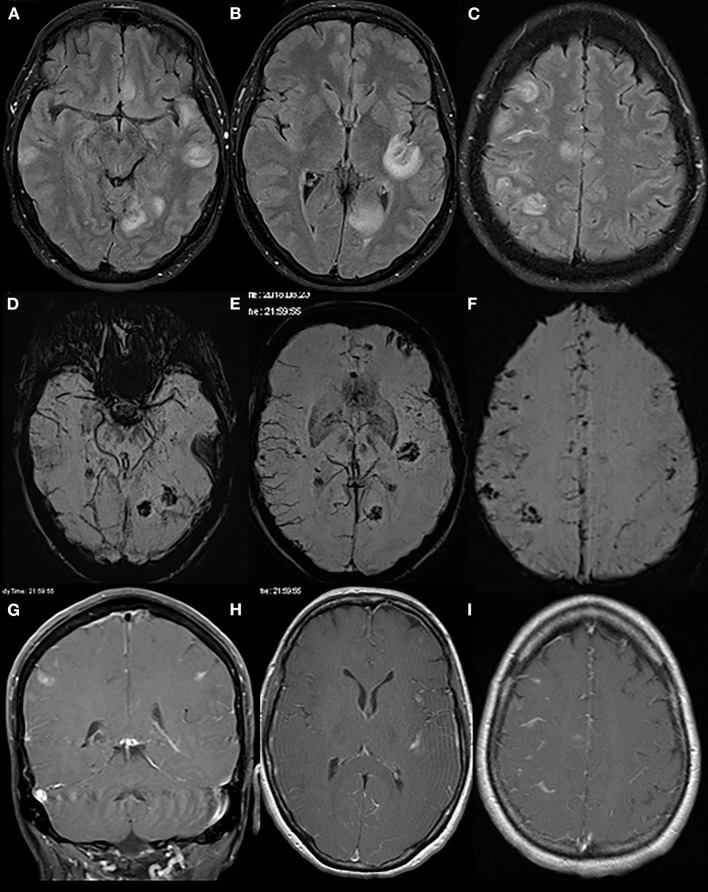
Initial brain MRI. Brain MRI displays multiple round/ovoid cortico-subcortical lesions with surrounding edema (T2-FLAIR sequences: **A–C**). Some lesions had a mainly central hemorrhagic component (susceptibility-weighted imaging, **D–F**). On contrast-enhanced T1 scans **(G–I)**, there was partial lesional and leptomeningeal contrast enhancement.

An initially performed CT of the brain (CCT) revealed a newly formed hypodensity in the left occipital region, which was not present on an archival CCT scan from the year before. A subsequently performed MRI of the brain furthermore revealed multiple, contrast-enhancing lesions with central hemorrhagic transformation and slight edema formation predominantly at the cortico-medullary border in both hemispheres along with an increased leptomeningeal contrast enhancement (Figure 1). From this picture, a septic embolic disease was initially suspected; cardiologic evaluation including transthoracal echocardiography however did not show any signs of endocarditis. Sonography of the intra- and extracranial arteries was unremarkable as well. Apart from an insignificant elevation of C-reactive protein and creatine kinase, routine blood analysis was normal.

The patient was admitted to our neurological intensive care unit (ICU) and an empiric antibiotic therapy was started consisting of ceftriaxone, metronidazole, and rifampicin. Clinically, over the following days, the patient developed a progressive delirium along with dyscognitive seizures with speech arrest, which were treated with sedatives (propofol, dexmedetomidine, and lorazepam) and valproic acid, respectively. At that time, the EEG however was normal.

CSF analysis at admission showed a lymphocytic pleocytosis of 124 cells/μl (93% lymphocytes, 7% monocytes) with a normal lactate of 1.9 mmol/l and an elevated level of protein content of 112 mg/dl. There was a moderate blood–brain barrier dysfunction (albumin quotient 23.32) and a pathologically elevated IgM index of 0.69; oligoclonal bands were not detectable. Extended laboratory tests for infectious disease in both CSF (borrelia, toxoplasmosis, adenovirus, EBV, enterovirus, herpes simplex I and II, varicella zoster virus, FSME) and blood (aerobic and anaerobic blood cultures, immunopathological screening tests for irregular antibodies, *Aspergillus, Candida albicans, Bartonella henselae, Toxoplasma gondii, Coxiella burneti*, tuberculosis, HIV) came back as negative. Serum for testing of autoimmune encephalitis parameters (anti-neuronal and anti-glial antibodies) was sent to the respective specialized lab; however, results were not immediately available.

As autoimmune encephalitis was soon clinically suspected, high-dose i.v. steroids (methylprednisolone 500 mg once daily) were administered already on day 2 after admittance, while the antibiosis was continued. However, the patient continued to deteriorate, appeared slow with decreased consciousness and fluctuating intense headache episodes. Repeated EEGs also showed a progressive slowing, first with a punctum maximum in both frontal lobes, then generalized over the full brain. A control CT scan of the brain on day 5 after admittance to the hospital consecutively revealed a massive progression of a generalized brain edema. Intravenous steroid dose was increased to 1 g methylprednisolone once daily i.v. along with osmotic therapy to treat the edema (mannitol 15% 125 ml i.v. 4x/day, Acetazolamide 250 mg i.v. 2x/day, hypertonic saline); however, disease further progressed. On day 7, the patient suddenly presented with a comatose state, wide and dilated pupils unreactive to light and positive pyramidal signs along with decerebrate rigidity.

Immediate intubation was required. Brain death was diagnosed on day 8 after admittance.

Only after death of the patient the lab report of serum MOG-Abs (assay using full-length MOG C-terminally fused with EGFP; titer 1:320) was available. Serum anti-aquaporin 4 and anti-glycine receptor antibodies as well as antibodies against NMDAR, CASPR2, AMPAR1/2, LGI1, DPPX6, and GABA (B) R in serum were negative.

## Neuropathology

**Figure 2:**
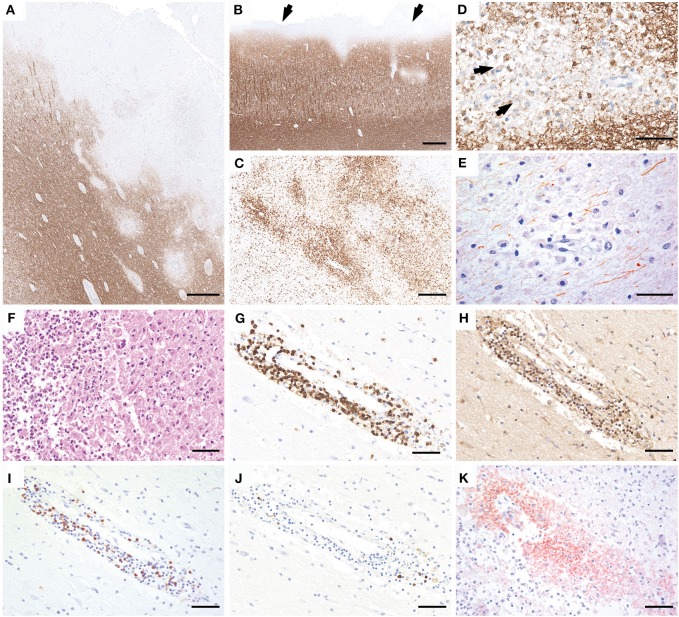
Neuropathology of brain autopsy. Histopathology of the brain autopsy reveals numerous, predominantly cortical demyelinating lesions that expand into the subcortical white matter (**A**; MOG) or form a subpial band of demyelination (**B**, arrows). The lesion borders show numerous activated microglia and macrophages (**C**, HLADR). The plaques are characterized by partly confluent, partly perivenous areas of demyelination, with MOG-positive myelin degradation products within the macrophage cytoplasms (**D**, arrows). Axons are relatively better preserved but the density is clearly reduced compared to the peri-plaque white matter (**E**, SMI31). Some lesions show superimposed ischemic damage with tissue necrosis with pronounced infiltration of neutrophilic granulocytes (**F**, H&E). The inflammatory infiltrates are mainly composed of CD3 **(G)** and CD4-positive T cells **(H)**, less CD8-positive T cells **(I)**, and few perivascular CD79a-positive B cells **(J)**. Within the lesions, profound perivenous deposition of activated complement complex is visible (**K**, C9neo antigen). Scale bars: **(A,B)** 600 μm; **(C)** 300 μm; **(D,E)** 50 μm; **(F–K)** 60 μm.

Gross examination of the brain showed severe edema with grayish discoloration of the gray and white matter. The spinal cord was not investigated. Histological examination of frontal, temporal, parietal, and occipital lobes; hippocampus; basal ganglia; brainstem; and cerebellum revealed multiple, predominantly cortical demyelinating lesions in the frontal, temporal, and parietal lobe with intracortical, leukocortical (Figure 2A), and subpial plaques (Figure 2B). The lesions were composed of partly confluent, partly perivenous areas of demyelination in the gray and white matter (Figures 2A,B). The lesion borders were characterized by a rim of numerous activated microglia and macrophages (Figure 2C) with early active demyelination activity with Luxol fast blue- and MOG-positive myelin degradation products within macrophage cytoplasms (Figure 2D). Axons within the lesions were relatively better preserved but showed a pronounced reduction of axonal density in comparison to the periplaque white matter (Figure 2E). Some demyelinated areas showed a superimposed ischemic damage with tissue necrosis with loss of astrocytes and axons and infiltration of neutrophilic granulocytes (Figure 2F). These necrotic areas were negative for fungi or bacteria. The inflammatory infiltrates in the demyelinated plaques mainly contained CD3- and CD4-positive T cells (Figures 2G,H), less CD8-positive T cells (Figure 2I), and only few perivascular CD20- and CD79a-positive B cells (Figure 2J). Active demyelination was associated with pronounced perivenous deposition of activated complement complex (C9neo antigen) (Figure 2K).

## Discussion

Acute inflammatory demyelinating diseases of the CNS comprise heterogeneous entities such as multiple sclerosis (MS), neuromyelitis optica spectrum disorders (NMOSD), and acute disseminated encephalomyelitis (ADEM), varying in pathogenesis, disease course, and severity as well as prognosis and associated biomarkers (1). The presence of MOG-Abs is predominantly found not only in children with CNS demyelinating diseases but also in adults with ADEM and aquaporin 4 antibody-negative NMO cases (2, 3) and in patients with NMDAR encephalitis with demyelination (4).

The clinical presentation of MOG-Abs-associated disorders usually changes with the patients' age; in young children, MOG-Abs are associated with an ADEM-like presentation, while adults usually present with a predominant involvement of optic nerves and the spinal cord (5).

Usually, the presence of anti MOG-Abs indicates a more favorable prognosis with a good response to steroid therapy, although a subset of patients may develop persistent neurological deficits mainly as sequelae of the first attack. Recently, a case of a fulminant clinical course with predominant optic and spinal cord involvement in a 71-year-old male requiring intensive care treatment within a few days after onset and a fatal outcome within 4 months despite aggressive immunotherapy has been reported (6). This patient had overlapping MOG and aquaporin 4 antibodies, and histopathology revealed features of acute MS pattern II without any cortical involvement.

Cortical involvement in MOG-Abs-positive adult patients (age range 23–39 years) has been described in a few cases. The main clinical presentations were generalized epileptic seizures with or without abnormal behavior and consciousness disturbances (7). After high-dose methylprednisolone, unilateral cortical FLAIR T2-hyperintense lesions disappeared and patients fully recovered. Unfortunately, no histopathological data are available on these cases. Ikeda et al. described a similar case of a 29-year-old female presenting with generalized seizures, unilateral cerebral cortical lesions, and a bilateral optic neuritis, who underwent a brain biopsy before steroid treatment. This revealed only mild inflammatory changes in the cortex and subcortex without distinct demyelination (8) and did not fulfill the previously described MOG-Abs-associated histopathological features (9–13). The authors ascribe this to the most likely very early stage of demyelination, when the biopsy was taken. This patient also made a full recovery after steroid treatment.

Here, we report a fulminant fatal course of a 52-year-old patient positive for anti-MOG-Abs with predominantly cortical demyelinating lesions in the frontal, temporal, and parietal lobe irresponsive to high-dose steroid treatment. There was no clinical sign indicating spinal cord or optic nerve involvement.

In line with previously published neuropathology of MOG-Abs-associated demyelination, our case did show well-demarcated partly confluent, partly perivenous plaques, relative preservation of axons, and numerous macrophages and complement deposition, compatible with demyelinating features of MS pattern II (14). In addition, some areas showed superimposed ischemic tissue damage with necrosis, loss of astrocytes and axons, and infiltration of neutrophilic granulocytes, most likely due to reduced perfusion in the context of the severe brain edema. In line with most MOG-Abs-associated demyelinating diseases, a marked CSF pleocytosis was present while oligoclonal bands were negative. Our case expands the histopathology of MOG-Abs-associated demyelination to predominantly cortical demyelination. The older age of the patient together with the lesion distribution may have contributed to the unusually aggressive disease course and fatal outcome in this case.

## Data Availability Statement

The datasets generated for this study are available on request to the corresponding author.

## Ethics Statement

Written consent was obtained from the deceased patient's spouse for the publication of any potentially identifiable images or data included in this article.

## Author Contributions

SH wrote the manuscript and was involved in treating the patient. TG was involved in treating the patient as well as neuroradiological evaluation. MA and VS performed the autopsy. MH performed the immunohistochemistry of the brain specimen. CE performed the neuroradiological evaluation, discussion of results. RH analyzed the brain tissue samples and evaluated the neuropathological specimen.

### Conflict of Interest

The authors declare that the research was conducted in the absence of any commercial or financial relationships that could be construed as a potential conflict of interest.
